# Flawed methods explain the effect of mammography screening in Nijmegen

**DOI:** 10.1038/bjc.2011.264

**Published:** 2011-07-26

**Authors:** K J Jørgensen

**Affiliations:** 1 The Nordic Cochrane Centre, Rigshospitalet, Department 3343, Blegdamsvej 9, Copenhagen DK-2100, Denmark

**Sir,**

van Schoor *et al* (2011) apply a case–control design to evaluate the Nijmegen breast screening programme, which began in 1975. They estimate a 65% reduction in breast cancer mortality during the period 1992–2008 by calculating the odds ratio for the breast cancer death rate for screening participants *vs* non-participants. It is not the first time that this study design has been used to claim a large effect of screening mammography in Nijmegen (Verbeek *et al*, 1984). In 1984, some of the same authors used a similar design to claim a 52% reduction in breast cancer mortality by 1981. This large effect was surprising, as the same authors reported that there was no actual reduction in the breast cancer mortality rate in Nijmegen at that time, when participants and non-participants were combined (Verbeek *et al*, 1984). Interestingly, although the new study covered a longer observation period (1975–1991), it identified an effect of only 28% in Nijmegen by 1991 (van Schoor *et al*, 2011). No explanation for this discrepancy was provided, although the title of their new study emphasises the ‘increasingly strong reduction in breast cancer mortality due to screening over time’ (van Schoor *et al*, 2011).

The case–control design has been shown to provide severely flawed results when estimating the effect of mammography screening programmes, with specific reference to the 1984 study of Nijmegen (Janzon and Andersson, 1991). The investigators of the Malmö randomised screening trial elegantly demonstrated how large the bias can be. They compared breast cancer mortality rates in participants *vs* non-participants within the screening arm of their trial (Janzon and Andersson, 1991). After 9 years, by the end of 1986, the relative risk for breast cancer mortality was 0.96 (95% CI 0.68–1.35; Table 1) when the trial was analysed as a randomised trial (Janzon and Andersson, 1991). But when the authors used the case–control design of Verbeek *et al* (1984) and van Schoor *et al* (2011), they found a significant (but false) 58% ‘effect’ (OR matching for age; 0.42, 95% CI 0.22–0.78; Table 2; Janzon and Andersson, 1991).

This impressive but spurious ‘effect’ in the Malmö trial was attributed to selection bias (Janzon and Andersson, 1991). Some women decide not to go for screening because they fear something may be wrong, and women from lower socioeconomic groups also often choose not to be screened, but have higher mortality from a range of diseases, including breast cancer. The Malmö trial authors concluded that we must therefore compensate for selection bias if we apply the case–control design to evaluations of public health effects of mammography screening. They also demonstrated that the arguments against the importance of selection bias previously presented by Verbeek *et al* (1984) were flawed (Janzon and Andersson, 1991). Verbeek *et al* (1984) compared incidence rates in Nijmegen with those in the unscreened area of Arnhem close by to argue that selection bias was unimportant. The obvious comparison would have been the breast cancer mortality rates in these two areas, but this was not done and would likely have provided unfavourable results, as breast cancer mortality rates in Nijmegen were not actually reduced. As the Malmö trial found no effect by 1986 (an effect only appeared later), whereas the ‘effect’ was 58% when the case control design was used, the 65% ‘effect’ found by van Schoor *et al* (2011) is unimpressive.

Many of these concerns were also raised in the 2002 WHO/IARC report (Vainio and Bianchini, 2002) in its evaluation of case–control studies of mammography screening, including again specifically the study by Verbeek *et al* (1984), which led them to conclude that, ‘observational studies based on individual screening history, no matter how well designed and conducted, should not be regarded as providing evidence of an effect of screening’ (Vainio and Bianchini, 2002).

Recent studies with much stronger designs that compare screened with non-screened areas within the same countries over the same time period have not found an effect of current mammography screening programmes (Jørgensen *et al*, 2010; Kalager *et al*, 2010). The Norwegian study (Kalager *et al*, 2010) was criticised because the average follow-up of 2.2 years was considered too short, but this is a misunderstanding. This was the follow-up after diagnosis. The follow-up from start of screening was 6.6 years, which is when an effect was seen in the randomised trials.

## Figures and Tables

**Table 1:** The Malmö mammographic screening trial assessed as a randomised trial (Janzon and Andersson, 1991)

	**Invited (*n*=21 088)**	**Controls (*n*=21 195)**
Proportion	50%	50%
Breast cancer deaths	63 (31 were non- participants)	66
Mortality rate	0.299%	0.311%
Relative risk	0.96 (95% CI 0.68–1.35)	

**Table 2:** The Malmö trial assessed using a case-control design Janzon and Andersson, 1991)

	**Living controls (random sample)**	**Women dead from breast cancer**	**Total**
*Participation in screening*			
Yes	229	36	265
No	71	24	95
Total	300	360	
Crude odds ratio	0.46		
Adjusted (matching for age) odds ratio	0.42 (95% CI 0.22–0.78)		
